# A method for the identification of COVID-19 biomarkers in human breath using Proton Transfer Reaction Time-of-Flight Mass Spectrometry

**DOI:** 10.1016/j.eclinm.2021.101207

**Published:** 2021-11-20

**Authors:** Aikaterini Liangou, Antonios Tasoglou, Heinz J. Huber, Christopher Wistrom, Kevin Brody, Prahlad G Menon, Thomas Bebekoski, Kevin Menschel, Marlise Davidson-Fiedler, Karl DeMarco, Harshad Salphale, Jonathan Wistrom, Skyler Wistrom, Richard J. Lee

**Affiliations:** 1 RJ Lee Group Inc., Monroeville, PA, USA; 2 Edelweiss Technology Solutions LLC, Novelty, OH, USA; 3 Mercyhealth, Janesville, WI, USA; 4 Henry Ford Health System, Detroit, MI, USA; 5 QuantMD, Pittsburgh, PA, USA; 6 Department of Bioengineering, University of Pittsburgh, Pittsburgh, PA, USA; 7 Practical Sustainbility LLC, Maryville, MO, USA

## Abstract

**Background:**

COVID-19 has caused a worldwide pandemic, making the early detection of the virus crucial. We present an approach for the determination of COVID-19 infection based on breath analysis.

**Methods:**

A high sensitivity mass spectrometer was combined with artificial intelligence and used to develop a method for the identification of COVID-19 in human breath within seconds. A set of 1137 positive and negative subjects from different age groups, collected in two periods from two hospitals in the USA, from 26 August, 2020 until 15 September, 2020 and from 11 September, 2020 until 11 November, 2020, was used for the method development. The subjects exhaled in a Tedlar bag, and the exhaled breath samples were subsequently analyzed using a Proton Transfer Reaction Time-of-Flight Mass Spectrometer (PTR-ToF-MS). The produced mass spectra were introduced to a series of machine learning models. 70% of the data was used for these sub-models’ training and 30% was used for testing.

**Findings:**

A set of 340 samples, 95 positives and 245 negatives, was used for the testing. The combined models successfully predicted 77 out of the 95 samples as positives and 199 out of the 245 samples as negatives. The overall accuracy of the model was 81.2%. Since over 50% of the total positive samples belonged to the age group of over 55 years old, the performance of the model in this category was also separately evaluated on 339 subjects (170 negative and 169 positive). The model correctly identified 166 out of the 170 negatives and 164 out of the 169 positives. The model accuracy in this case was 97.3%.

**Interpretation:**

The results showed that this method for the identification of COVID-19 infection is a promising tool, which can give fast and accurate results.

**Research in context**

**Evidence before this study**

COVID-19 infection may lead to specific changes in the volatile organic compounds (VOCs) pattern of the exhaled breath, thus providing a unique diagnostic tool. Proton transfer reaction-mass spectrometry has been shown to be a reliable tool in identifying VOC concentrations at ultra-low concentrations in highly complex matrices, such as breath analyses for various applications.

**Added value of this study**

An algorithm has been developed based upon the mass spectrometric analysis of 1000+ breath samples from different sources with different ambient backgrounds. This algorithm has been shown to predict fairly well the status of patient in regard to being identified as COVID-19 positive with PCR or not. Specific biomarkers have been identified to be correlated to the infection with SARS-CoV-2, and it has been identified that these biomarkers are age related.

**Implications of all the available evidence**

The COVID-19 status of a person can be identified using a breath sample collected in a sampling bag. The situation of sampling, i.e., the VOC background is of lesser importance than the knowledge of age of the patient. The VOC background does not necessarily have to be identified and subtracted from the spectra prior to analysis. This opens the ability to use the approach in a direct sampling strategy without sampling bags for quick screening. Comparative research needs to be done to identify which of the biomarkers are unique to a COVID-19 infection as opposed to general infections of the vascular system.

Alt-text: Unlabelled box

## Introduction

SARS-CoV-2, the virus causing the illness known as COVID-19, has caused a pandemic the likes of which the world has not seen in over 100 years. While the world and its technology have changed substantially in that time, human physiology has not. The World Health Organization (WHO) [1] has reported over 190 million cases, and over 4 million deaths from this disease, as of July 29^th^ of 2021. The world has relied heavily on administrative and physical measures such as social distancing, mass testing, and quarantining procedures to try to slow the spread of this disease [2]. With record breaking speed, vaccines have been developed and distributed [3]. These measures are all important, but they still leave gaps in combating the pandemic. The aim of this study was to employ a novel approach using breath analysis. This would result in a large reduction in invasive measures during sampling compared to nasal swabs and significantly higher throughput rates.  We combined artificial intelligence and machine learning with one of the world's most sophisticated gas analyzers to develop a real-time profile of the breath from individuals infected with SARS-CoV-2 resulting in a paradigm shifting mass screening tool.

The analysis of volatile organic compounds (VOCs) in human breath holds valuable clinical potential and has been the subject of many research studies [4]. Testing for volatile biomarkers in clinical breath samples offers an option for developing rapid and potentially inexpensive disease screening tools with multiple advantages

1) Sampling can be readily repeated and is non-invasive.
2) Sampling and analyses can be done within a minute.
3) Allows to immediately start preventive measures such as isolation, use of Personal Protective Equipment (PPE) etc.

Over the last decade, multiple studies focused on the use of breath testing for the early diagnostics of acute respiratory distress syndromes (ARDS) and medication response [[5], [6], [7]].

A multitude of breath analysis studies related to viral infections in humans have been published. These include the human rhinovirus, Influenza A and the Influenza H1N1. The biomarkers correlated to these viral infections include 2,3-butandione, aldehydes, 2,8-dimethyl-undecane, and n-propyl acetate [8]. A specific study aiming at VOC emissions from cell cultures with human respiratory viruses resulted in the identification of a different suite of biomarkers, including acetone, 2-propanol, o-xylene, benzaldehyde, and benzonitrile, as well as the aldehydes 2-butenal, 2-propenal, 3-methyl-butanal, acetaldehyde, alkylated aldehyde, benzaldehyde, hexanal, nonanal, and propanal; in addition, the three furan derivatives furan, 2,3-dihydro-furan, and tetrahydrofuran were identified, but these were also related to bacterial infections [9]. The volatile emissions from Influenza virus infections have been published in multiple studies and include compounds such as acetaldehyde, propanal, acetone, and n-propyl acetate amongst others [10,11].

Several studies have been focused on the detection of COVID-19 patients using various techniques for breath analysis [12]. Grassin-Delyle and colleagues [13] presented a study using a PTR-MS (Proton Transfer Reaction Mass Spectrometer) and 40 patients with ARDS, with 28 being confirmed COVID-19 cases. The age of the non-COVID-19 patients ranged from 54 to 79 years old, while the COVID-19 patients were 55 to 72 years old. Using a multivariate analysis, they were able to develop a method with an accuracy of 93% (sensitivity: 90%, specificity: 94%). The four most prominent volatile compounds in COVID-19 patients were methylpent-2-enal, 2,4-octadiene 1-chloroheptane, and nonanal. Shan and colleagues [12] presented a breath analysis study using a nanomaterial-based sensor array. The device included eight 1 mm diameter sensors with specific organic functionalities targeting dodecanethiol, 2-ethylhexanethiol, 4-tertmethylbenzenethiol; decanethiol; 4-chlorobenzenemethanethiol, 3-ethoxythiophenol, tertdodecanethiol, and hexanethiol.  Within this study, 49 confirmed COVID-19 patients, 58 healthy controls, and 33 non-COVID lung infection controls were tested.  The mean age of the patients was 59 years, with 57% females. The tested groups were separated in training sets (70% samples) and test sets (30% samples). The training and test set data showed an accuracy of 94% and 76% respectively, in differentiating patients from healthy individuals. In addition, the device showed a 90% and 95% accuracy in differentiating between patients with COVID-19 and patients with other lung infections.

Multiple studies presented the use of gas chromatography-ion mobility spectrometry (GC-IMS) for the breath analysis [[14], [15], [16], [17]]. Ruszkiewicz and colleagues [16] presented a study in which ninety patients from Edinburgh, UK (65 patients, 10 positive COVID-19 cases) and Dortmund, Germany (25 patients, 17 positive COVID-19 cases) were tested. The non-COVID -19 cases were suffering from other respiratory diseases, such as asthma, COPD, and bacterial pneumonia or cardiac diseases. The accuracy of the method for the identification of the COVID-19 patients was 80% and 81.5% in each group, respectively. The biomarkers that worked as discriminants for the Edinburgh study were ethanal, acetone, 2-butanone, acetone/2-butanone cluster, methanol monomer, methanol dimer, and octanal. The distinct biomarkers for the Dortmund group were ethanal, acetone, 2-butanone, methanol monomer and dimer, and heptanal.

These studies focused solely on identifying a pattern within hospitalized patients. While this is the first step to identify a pattern of COVID-19 within the exhaled breath, it also has several downsides:

1) The concentrations of breath biomarkers of hospitalized patients may change due to the clinical environment in which they are isolated.
2) Medications are known to have severe impacts on the metabolism of a patient and can be directly correlated to changing breath patterns.
3) These cases are of a severity that is not representative of the general population.

While the impact of the first two aspects has been studied in detail by Trefz and colleagues [18], the latter aspect has specific implications from an analytical perspective when targeting people that feel healthy but are infected by SARS-CoV-2.

Given the work that has been previously done, our study attempts to maintain scientific integrity while allowing for the exposure of the subject in VOCs that are common in ambient environments. 1137 patients with an age range from 3 to 96 were studied. The studies involved multiple locations and included hospitalized COVID-19 positive patients for a direct comparison of the data. Breath results were compared to the results of the person's traditional COVID-19 test by Polymerase Chain Reaction (PCR) or Nucleic Acid Amplification Test (NAAT). Breath was analyzed using Proton Transfer Reaction Time-of-Flight Mass Spectrometer (PTR-ToF-MS). Each breath pattern (breath print) was then analyzed by a variety of machine learning algorithms against their PCR or NAAT COVID-19 test results to identify a specific physiologic change pattern associated with COVID-19. To our best knowledge this is the largest study of its kind to date.

## Methods

### Location

This study used two locations of varying population demographics. Both arms underwent individual IRB approval. The first arm was performed at the Mercyhealth North Emergency Department in Janesville, WI (MH-study). The MH-study took place from 08/26/2020 till 09/15/2020. A total of 955 samples were collected at a drive-through COVID-19 testing station and the emergency department itself. All donors were sampled and tested for COVID-19 using a PCR test (943) or a NAAT (12). The second arm was located within the Henry Ford Health System in the greater Detroit area, MI (HF-study) and was performed from 11/09/2020 till 11/11/2020. The HF-study was focused on 182 hospitalized patients using the same sampling and analytical techniques. The goal of the HF-study was to gather a comparative data set to the aforementioned breath analysis studies and to identify a pattern within COVID-19 positive hospitalized patients. All 182 hospitalized patients had positive PCR test results.

Both studies fall within the US Department of Health and Human Services (HHS) region 5. Based on the seasonality of influenza within this region, the corresponding interference by influenza-based infections on the breath analysis is therefore very limited in the case of the MH-study (week 36), while the HF-study (week 46) is on the onset of the seasonal peak. Therefore, the MH-study data represent a unique dataset insofar that COVID-19 cases were present, but the influenza rate was at its lowest in the season. This observation is based on the publicly published data by the Center for Disease Control's Fluview program (https://gis.cdc.gov/grasp/fluview/fluportaldashboard.html).

### Sample collection

The MH study was focused on collecting as many samples as possible in order to have enough information of the characteristic biomarkers in COVID-19 subjects that will allow a successful model development. After the MH study was completed the first version of the algorithm was developed which showed the need for more positive samples in order to have a good characterization of the COVID-19 positive footprint. Thus, the second study took place in Henry Ford Hospital to collect samples of patients with a higher severity of the disease. In this study, 182 positive samples were collected.

All samples were obtained in an informed consent fashion with limited HIPAA release, as reviewed and approved by IRB, allowing researchers to access medical records for traditional SARS-CoV-2 results. Mercyhealth's IRB number is 00004155. Henry Ford Hospital's IRB number is 14234. No lower age limit was included in the Mercyhealth arm. Consent was obtained from all patients and in the case of pediatric patients consent was obtained from a parent or legal guardian.

The subject was asked to drink a mouthful of water prior to collecting a breath sample to possibly reduce any VOC contamination from food and drink. This procedure has been found to be beneficial in removing the contributions of oral compounds to the breath sample, such as volatile fatty acids, aldehydes and phenols [19]. Each sample was collected from the donor in a 1L TEDLAR® bag. At MH study the subjects remained in their vehicle inside the drive-thru garage. The sampling was done in patient rooms in the HF study. All sample collectors used personal protective equipment (PPE). The subject was blowing into a 1-foot long ¼” diameter Perflouroalkoxy (PFA) tube attached to the bag, until the bag was fully inflated. The TEDLAR bag filling included more than one exhalation. For the kids the attending resident and the parent helped turning on and off the valve between exhalations to avoid contamination of the samples. After the sample collection the bag was sealed with the integrated bag valve, and eventually analyzed with the PTR-ToF-MS. The criteria of rejecting a sample were insufficient volume of sample and/or contamination while sampling which could have been resulted by wrongful turn on/off of the bag inlet valve. The samples that were falling into these rejecting criteria were excluded from the analysis. A PCR swab or an antigen test were also collected.

While sampling using bags is convenient for multiple reasons, such as speed of sampling, ease of logistical setup and ease of use by the patients, it has multiple downsides, including the potential for diffusion of compounds. This diffusion can happen in both directions, so sampled breath can lose compounds and exterior air compounds can diffuse into the bags. The speed and extent of such exchange is highly dependent on the bag material, the conditions of storage of such bags and the duration of storage of the sample within these bags [[20], [21], [22]]. In a preliminary study, we compared three different categories of available bags, TEDLAR®, a multilayer foil gas sampling bag and an ALTEF gas sampling bag. TEDLAR® bags were found to have the least impurities compared to the other two with no statistically sound difference in gas exchange from the environment over the period of multiple hours (Fig. S1 and Fig. S2).  Therefore, TEDLAR® bags were chosen for this study.

The MH-study lasted 21 days and 955 patients were tested; 88 symptomatic positive samples, 27 asymptomatic positive samples and 840 negative samples. The daily number of samples that got collected and analyzed varied and depended on the patients that were coming to get tested. The maximum of samples analyzed in one day was 150. Each sample was analyzed within minutes. During the HF study, which lasted 3 days, 182 symptomatic positive samples were collected. The samples were collected and delivered for analysis in batches of ten. The maximum time between the sample collection and the sample analysis was 2 hours. Overall, most of the samples were analyzed within 1 hour of their collection for both studies.

### Sample analysis and mass spectra deconvolution

The sample analysis was performed using a PTR-ToF-MS (Ionicon, PTR-TOF 4000) (Fig. S3). The PTR-ToF-MS is a high resolution and high sensitivity continuous real-time monitoring instrument that measures VOCs based on a soft ionization technique and subsequent mass spectrometry [23]. More specifically, the sampled air gets continuously drawn into the reaction chamber, where the sample gets mixed with a stream of pre-made hydronium ions. Based on its proton affinity, each volatile compound can get charged (i.e., protonated) or not. The ionized compounds are then introduced in the mass spectrometer, where they are separated and identified by mass. The un-protonated remainder of the air gets released by the instrument. Major compounds of air, such as nitrogen or oxygen, do not get ionized and are therefore not contributing to the background.

The key variables of the analysis are the volume of air drawn into the instrument over a period of time, the temperature of the sample inlet, and the temperature, pressure and voltage applied in the reaction chamber. The temperature, the pressure and the voltage of the reaction chamber control the ionization reaction rate, allowing for more or less volatile chemical compounds to be protonated, therefore directly impacting the detection limit of the analysis. These parameters also control the fragmentation rate of the targeted compounds. Fragmentation of molecules creates very specific patterns in the mass spectrometer for each compound. Enhanced fragmentation however, such as at higher voltage settings, can lead to a less trivial data analysis of the mass spectrum.  The overall suitability of soft ionization techniques, like the PTR-ToF-MS, for clinical breath analysis has been reported by Trefz and colleagues [18].

During the sample analysis, the PTR-ToF-MS was using hydronium as the primary reagent ion. The flowrate was set at 100 mL/min and the inlet temperature was at 80°C. The reaction chamber pressure, temperature and voltage were at 2.2 mbar, 70°C and 600V, respectively. The instrument was calibrated daily, using a gas mixture standard. Each TEDLAR® bag inlet was connected to the PTR-ToF-MS inlet. The sample from the bag was introduced in the instrument for a duration of 200 seconds and the spectra were recorded at 1Hz. This allowed observations on the stability of the resulting spectrum. Based on repeated analyses of the bag contents, the bag sample can be assumed to be a well-mixed sample, allowing to average the mass spectra over a specific period within the 200 second sampling segments. The time period used was the last 100 seconds of the 200 second spectrum, based on the variations of contents within that period.

The mass spectra were analyzed using the instrument's internal software (Ionicon, PTR-MS viewer software v. 3.2.5). In addition to the instrument's automatic continuous mass calibration, a manual inspection step was performed to validate the mass calibration. High mass resolution data analysis was performed to identify the compounds of interest and to maximize the accuracy of the contribution of each compound to the total mass. This step is particularly important for untargeted analysis in which all the compounds, even the ones at low concentrations, need to be identified and quantified. During lower resolution analysis (1 amu), the presence of a compound at a high concentration, such as acetone, can cause interferences with the accurate identification and quantification of neighboring compounds. While the instrument performed the analysis in high mass resolution, this level of separation turned out to be of low importance to the accuracy of the final prediction model.  Therefore, the peak resolution was reduced to nominal masses during the post processing analysis.

The background ambient air from the MF study was tested to confirm that the PTRMS measured mass spectra were not contaminated by the garage testing area and the cars' emissions. Once the patients were getting in the garage area, they were turning their car engine off. The TEDLAR bags used for the sampling had an on-off valve. When the patients were exhaling in the bag the valve was on, while immediately after the sampling the valve was turned off to avoid contamination of the samples with ambient air. The VOCs that are associated with vehicle exhaust are benzene, toluene and their byproducts [25].We did not see these compounds being elevated during our testing nor were they identified as important compounds by the developed algorithm suggesting that the vehicle exhaust emissions effects were negligible.

### Model development

For the development of our method, we used the results from the negative PCR tested donors and positive symptomatic PCR/NAAT donors. The deconvoluted and adjusted spectral information was used to feed into a multiplex of modeling algorithms.

Several peaks were excluded from the evaluation, either because their contribution cannot be linked to a patient's metabolic status but rather to instrument's parameters or that they are directly linked to an instrument input such as the continuous mass calibration. More specifically, we used m/z 30, and m/z 40 to m/z 400. We did not use compounds less than m/z 30 since they mainly consist of compounds that are relevant to the reagent ions and their changes are not affected by the patient status, but they are affected by the instrument's operation parameters, such as the pressure, temperature, and voltage of the reaction chamber. Also, m/z 204, m/z 205, m/z 329, m/z 330, m/z 331 and m/z 332 are related to the instrument auto-calibration mechanism and were excluded. Acetone, m/z 59, was also excluded because it is a main product of a variety of oxidation processes in human breath and its large concentration (ppm levels) compared to the concentration of the other identified compounds (ppb level) was found to interfere with the algorithm results.

The initial development of the model did not involve the use of the m/z 30. High resolution analysis though showed that the mass of the ion contributing at m/z 30 was 29.995. This suggested that the compound was NO^+^. The NO^+^ signal is assumed to be due to the presence of nitroso compounds that fragment in the drift tube of the PTR-MS due to collision induced dissociation. Previous studies have shown the formation of nitroso compounds form in due to the activities of bacteria in the stomach [24]. For these reasons, the algorithm included m/z 30 in the analysis.

The study started with a high-resolution approach, since there was no indication from the information available at the time of study design (i.e., August 2020) whether the compounds of concern could be clearly identified using nominal masses alone. Since the AI model did not show any improvement when using the high-resolution data in both sensitivity and specificity of correctly identifying a PCR-positive individual, the high-resolution data were then summed to nominal mass data. The main benefit of having obtained the high-res data is that these could be used for post-analysis identification of compounds of concern, which would otherwise have not been possible. Figure S4 summarizes the overall process of developing models for datasets with a large number of unknown variables. The final step after the model building process is one that entails result representation through model dashboards, feature importance estimates for the training set, and different visualization methods such as prediction distributions or confusion matrices.  Models were optimized in terms of their operating point for minimizing per-class-error-rates in the training sets, therefore maximizing sensitivity and specificity in a balanced manner through an appropriate choice of probability threshold from receiver operator characteristics analysis.  Each model had a model-specific probability threshold for optimality in classification. We leveraged H2O (version 3.30.0.1) to host a multi-node cluster with a shared memory model to develop our final machine learning models with all computations conducted in-memory.

XGBoost Gradient Boosting Machines (GBM), H2O's Gradient Boosting Machines (GBM), Random Forests (Distributed Random Forests and Extremely Randomized Tree variety), Deep Neural Networks and Generalized Linear Models (GLM), were leveraged for the purpose of model building, each with its own hyperparameters that required optimization.  We searched for a universe of optimal binary classification models across five pre-specified XGBoost GBM models, a fixed grid of GLMs, a DRF, five pre-specified H2O GBMs, a deep neural network, an Extremely Randomized Trees (XRT) model, a random grid of XGBoost GBMs, a random grid of H2O GBMs, and a random grid of deep neural nets.

Training was conducted on a random fold of 70% of the data, selected using stratified random sampling of our dataset based on the response of COVID-19 class (i.e., PCR/NAAT positive or negative), with the remaining 30% left for out-of-sample testing. For the training, only samples from symptomatic positive subjects were used to capture as best as possible this difference in concentration between the compounds. It was assumed that the amplitude of some of the important for the COVID-19 identification compounds would be greater in the symptomatic individuals. The median age of the samples used for the training was 39 years old. Cross-validation, using 5-fold cross-validation, was conducted on the entire dataset for the purpose of establishing whether our trained models were overfit. Demographic and clinical information were not used for the model development.

For each algorithm, we identified which hyperparameters we consider to be most important, defined ranges for those parameters, and utilized random search to generate models. Since our models were developed using the H2O python library, the built-in Random Grid Search provisions that it includes were adopted.  A random combination of hyperparameters (sampled uniformly from the set of all possible hyperparameter value combinations) were tested instead of exhaustively testing all possible combinations for ranges of hyperparameters relevant to each model type. A stopping criterion was specified for when the random search would stop, based on a target accuracy to be achieved (i.e. defined in terms of a log-loss function based on the per-class error rates targeted). The range of recommended hyperparameter ranges comes from trial an error but also some guidance from H2O's model-specific user-manuals.

After training the base models, a Stacked Ensemble model was trained containing the best performing model from each algorithm family, i.e., one XGBoost GBM, Random Forest, Extremely Randomized Tree Forest, H2O GBM, Deep Learning, and a GLM model. The final ensemble was optimized for rapid inference in production use cases and included 5 base models, dropping the GLM model since it was not contributing significantly to the determination of the response of COVID-19 vis-a-vis the remaining models.

### Role of the funding source

The funding source participated in the study design, data collection, data analysis, data interpretation, writing of the report and decision to submit the paper for publication. All authors confirm they had full access to the data in the study and accept responsibility for the decision to submit for publication.

## Results

### Subject distribution

**Fig. 1:**
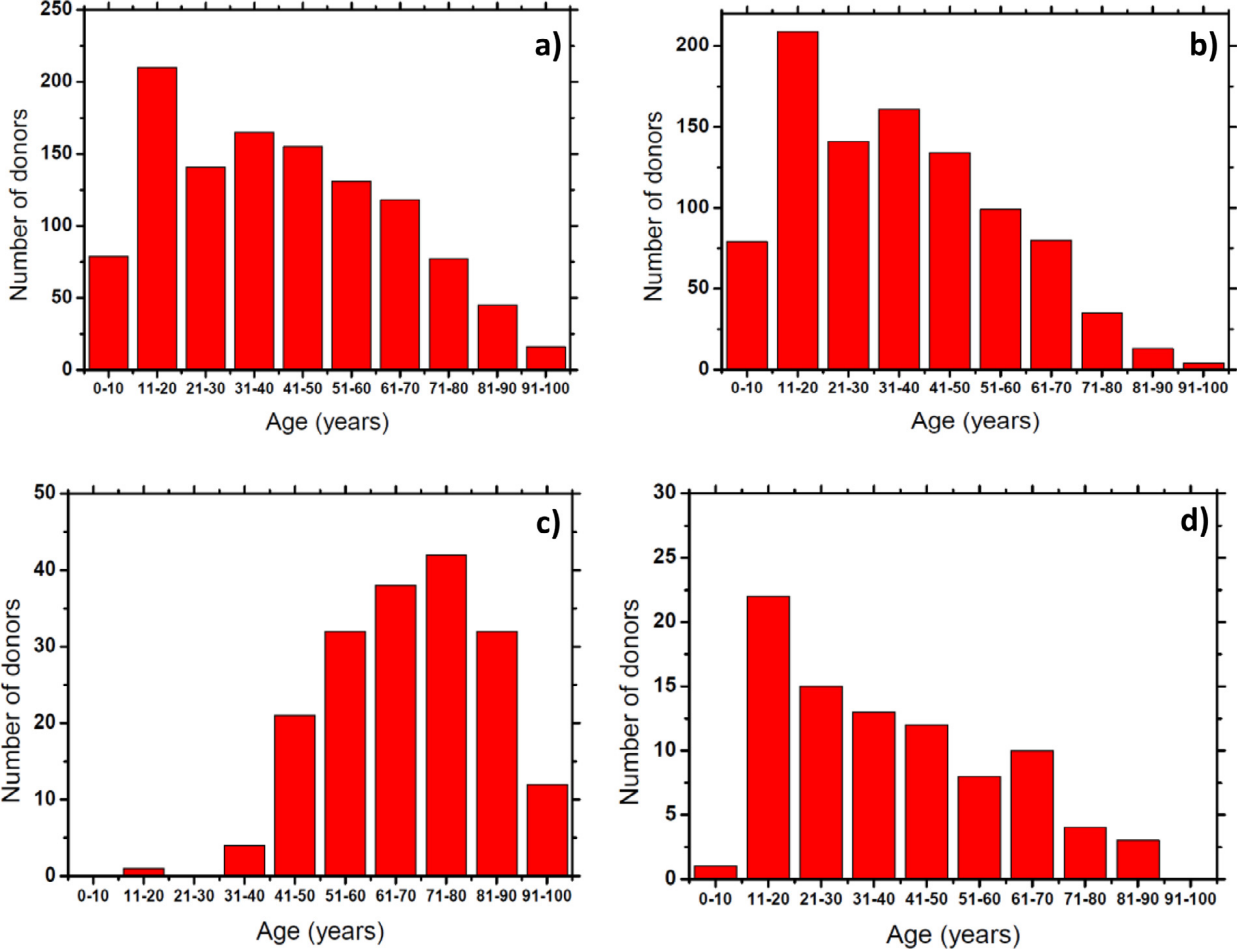
a) Total donor age distribution of samples collected during both studies. b) Total donor age distribution of samples collected during MH-study. c) Total donor age distribution of samples collected during HF-study. d) Donor age distribution of symptomatic positive COVID-19 donors collected during MH-study.

The subject age distribution is shown in Fig. 1 and Tables 1 and 2. The age range of tested subjects was between 3 and 96 years old, with most of the subjects being between 11 and 20 years old (Fig. 1a). For the MH-study the age range of the tested subjects was between 3 and 95 years old (Table 1), with most of the subjects having ages between 11 and 20 years old (Fig. 1b). The positive, symptomatic subjects from the MH-study ranged between 6 and 84 years old (Table 1), with most of them being around 11 to 20 years old (Fig. 1d). For the HF-study, the corresponding age range of the tested subjects of the total samples collected was between 19 and 96 (Table 2), with most of the subjects belonging to the 71 to 80 age range (Fig. 1c). This wide age distribution of the tested subjects allowed us for a better understanding of the effects of the COVID-19 in the breath biomarkers composition among different ages.

**Table 1:** Smoking and age status of the donors for MH study.

	Smoking status		Age (years)		Age Median (years)
	Smokers	Non smokers	Min.	Max.	
Symptomatic Positive	8	80	6	84	34
**Total**	87	868	3	95	34

**Table 2:** Smoking and age status of the donors for HF study.

Smoking status		Age (years)		Age Median (years)
Smokers	Non smokers	Min.	Max.	
10	172	19	96	69

Information regarding the smoking status and age of the donors was also collected. For the MH-study the percentage of smokers was 9%, while for the HF-study the corresponding percentage was 5.5%.

### PTR-ToF-MS results

**Fig. 2:**
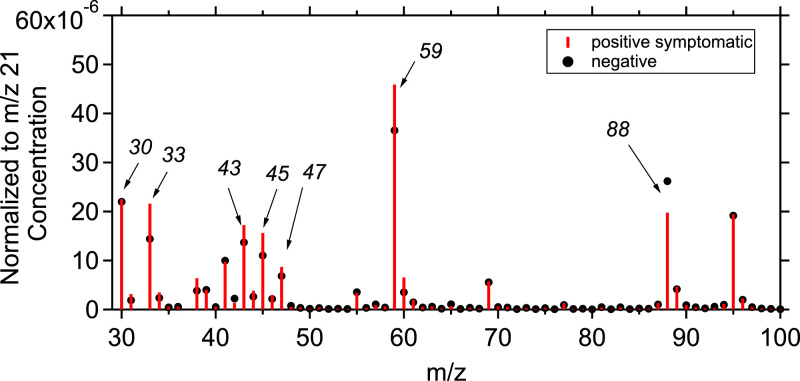
Mass spectra of the normalized (to the primary ion concentration at m/z 21) concentration (in ppb) of the total averaged positive samples of symptomatic donors (red bars) and the negative samples (black dots). The y-axis corresponds to the normalized concentration and the x-axis to the mass to charge ratios.

The average normalized to the primary ion (m/z 21) mass spectrum from the breath analysis of the total positive symptomatic patients was compared to the average mass spectrum from the breath analysis of the total negative samples. Fig. 2 shows the normalized concentration mass spectra for these samples for each m/z value, ranging from 30 to 100. The highest VOC concentrations were observed in m/z lower than 100, while the concentrations for m/z>100 were significantly lower. Figure S5 represents a heatmap of compounds with m/z <100 for the total samples collected showing the concentration differences between positive and negative samples. Acetone is the main product of all the oxidation processes and was found to have the highest concentration, at parts-per-million by volume (ppmv) levels. The rest of the compounds were at parts-per-billion by volume (ppbv) or in parts-per-trillion by volume (pptv) levels. When directly comparing the spectra, the concentration of compounds associated with m/z 88 was lower in the case of confirmed positive samples while m/z 33, m/z 45, m/z 47 and m/z 59 seem to be increased for these random samples. Figure S6 shows the percentage abundance of the important m/zs that found to have the biggest change for the positive and negative samples (according to the artificial intelligence algorithm). A comparison between the percentage abundance of the important m/zs between positive and negative samples for ages over 55 years old is also presented in Figure S7. In both Figures the biggest difference in concentration between positive and negative samples is shown in m/z 30, m/z 45 and m/z 47. It is clear though that the difference in these three compounds is not enough to determine if a sample is positive to COVID-19.

The analysis and the interpretation of the results showed that the determination of a sample being positive or negative to COVID-19 cannot be achieved just by an optical comparison of the mass spectra, since small patterns in concentration changes between the different m/z's cannot be easily identified. The algorithm used as inputs all the individual mass spectra from the patients. The developed artificial intelligence model has the capability to identify such patterns and account for such changes for the determination of a sample being positive to COVID-19.

### Model results

The mass spectra related to the PCR or NAAT positive and negative samples were introduced as inputs to the individual sub-models and were used for training and prediction. We combined 5 different sub-models for the determination of the most important compounds. Each sub-model contributed to the final prediction to determine if a sample is positive or negative to COVID-19. However, the importance of each model to the final prediction varies (Fig. S8). The first sub-model, Gradient Boosting, is the most important while the other ones have a smaller effect on the results.

In total, a set of 340 samples, 95 positives and 245 negatives, was used. 27 of the 95 positive samples were from asymptomatic subjects. The model successfully predicted 77 out of 95 samples as positives and 199 out of 245 samples as negatives. The overall accuracy of the model was 81.2%, the normal precision with respect to negatives was 91.7%, the specificity was 81.2% and the sensitivity was 81.1% (Table 3 and Table S1).

**Table 3:** Model predictions for all age groups

	COVID-19 predicted	Negative predicted	Prediction accuracy
**COVID-19 measured**	77	18	0.8105
**Negative** **Measured**	46	199	0.8122
**Total**	123	217	0.8118

The performance of the model for subjects over 55 years old was also tested. Santesmasses and colleagues [26] have shown that the COVID-19 fatality rate is higher to patients 55 years or older. The number of subjects was 339 of whom 170 were negative and 169 were positive. The model identified correctly 166 out of 170 negatives and 164 out of 169 positives. The accuracy of the model was 97.3%. This relates to 2.3% false negative and 2.9% false positive rate (Table 4 and Table S1). The accuracy of the model was, as expected, in favor of the >55 age category since over 50% of the total positive samples belonged in this category and used for both model training and testing.

**Table 4:** Model predictions for age 55+

	COVID-19 predicted	Negative predicted	Prediction accuracy
**COVID-19 measured**	164	5	0.9794
**Negative** **Measured**	4	166	0.9765
**Total**	168	171	0.9735

An additional model was developed which used the normalized to the primary ion (m/z 21) mass spectra for both training and testing. The same procedure and same sub-models were followed for the training of the model as in the base case. Approximately 70% of the total data which included symptomatic positive and negative samples were used for the model's training, while around 30% of the data (symptomatic positive, asymptomatic positive and negative) were used for the model testing. Table S2 summarized the model testing results. The model predicted 247 out of 247 negative samples, 29 out of 72 symptomatic positive samples and 0 out of 27 asymptomatic positives. The results showed that the use of the normalized mass spectra decreased the performance of the model compared to the base case.

A comparison of the prediction results between children and adults did not take place since not enough samples from children were collected in order to create trusted results that could be used for such a comparison.

The model identified the importance of each m/z on the determination of a sample as positive or negative to COVID-19. Each sub-model identified a different set of the most important m/z (Fig. S9).  Nitrogen oxide (m/z 30), butene (m/z 57), CH_4_S (m/z 48), acetaldehyde (m/z 45), heptanal (m/z 115), ethanol (m/z 47), a methanol water cluster (m/z 51), and propionic acid (m/z 75) were identified as important compounds for the identification of COVID-19 in human breath. Fig. S5 shows the abundance of the compounds of importance among the samples.

In the presented method m/z 45 has the second highest importance in the prediction. High resolution analysis of the samples has shown that on average CO_2_H+ accounted for 68% of m/z 45 while the rest was acetaldehyde. Interference of CO_2_H+ in the acetaldehyde signal has also been presented in previous studies [27]. M/z 45 (acetaldehyde) has been found to increase over 75% in the case of COVID-19 positive patients over 55 years old, with this percentage decreasing to 5% in the case of patients younger than 40 years old. (Table S3).

For octanal, a similar trend was found as with nonanal. The elevation is very distinct in older patients with a percentage increase of 44% in the case of COVID-19 positive patients. For patients less than 40 years of age, there was a slight decrease of 1% in concentration for infected patients in the case of octanal while in the case of nonanal the corresponding decrease was 10%. Heptanal, another aldehyde reported to be elevated in COVID-19 patients, also shows elevation, but lesser with increased age. More specifically for patients over 55 years old the increase was 7%, while for people less than 40% the corresponding increase was up to 39% (Table S3). Ketones, such as 2-butanone were increased around 53% in the case of COVID-19 positive patients older than 55 years old while for patients younger than 40 years old the corresponding increase was 6%. For 2-Pentyl-Furan, we found in older COVID-19 positive patients a 10% increase in concentration, on average. However, this changes for younger patients with ages <55 where 2-pentyl furan concentration was decreased by 10% (Table S3).

**Fig. 3:**
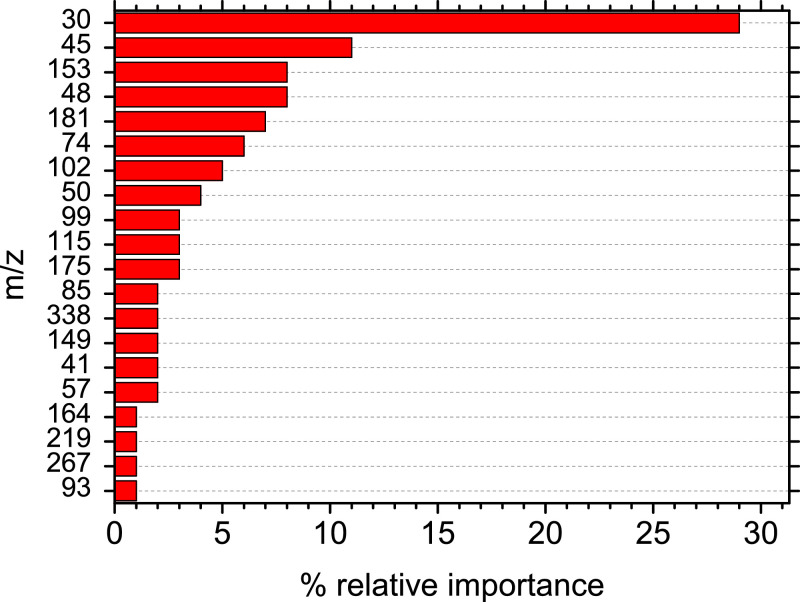
The 20 most important mass to charge ratios (m/z) for the prediction of a COVID-19 positive sample derived from the combination of the five sub-models.

The importance of each sub model together with the relative importance of each compound was combined in order to calculate the 20 most important compounds for the prediction of a COVID-19 positive sample. Fig. 3 shows that nitrogen oxide, CO_2_H and acetaldehyde are the compounds that affect the model's decision-making the most. More specific the relative importance of m/z 30 (nitrogen oxide) as it was calculated by the developed model was 27%, while the rest of the important compounds had a relative importance of 11% or lower (Figure 3). Also, m/z 30 (nitrogen oxide) appears to be the most important compound in all individual submodels with its contribution to the final decision being up to three times higher compares to the other compounds (Figure S9).

One compound that was also investigated was acetonitrile. Wzorek and colleagues [28] have supported the smoking-related origin of acetonitrile in the breath of smokers. The acetonitrile (m/z 42) was found to contribute less than 1% in the model prediction suggesting that the model prediction is not affected by the smoking status of the subjects. The model did not identify compounds specific to age groups. A future study will be focused more on the age depended COVID-19 biomarkers using methods like LIME and SHAP.

## Discussion

A method for the determination of a COVID-19 positive patient was developed by coupling the PTR-ToF-MS with an artificial intelligence algorithm. The method is based on the identification of COVID-19 biomarkers in breath using a non-invasive alternative to nasopharyngeal swabbing tests. TEDLAR bags were tested and identified to be the most suitable mean for the sample collection and its analysis using the PTR-ToF-MS. A total of 1137 different samples, 270 symptomatic positives, 27 asymptomatic positives and 840 negatives, were used to develop and test an algorithm that could predict the result of a breath sample in less than a minute. The entire model results had a sensitivity of 81% which is on par with many current rapid tests in the market. In the subset of patients over 55 the algorithm was more effective with a sensitivity and specificity of 97%. In the case of the over 55 years old algorithm, the same algorithm was used but only the samples over 55 years old were tested. This means that a percentage of the samples that were used for testing was also used for training purposes. The algorithm in its current form does not have the ability to identify which of the over 55 years old samples used for both training and testing.

In the prediction of the model, m/z 30, which relates to nitrogen oxide, was found to be of high importance. While nitrogen oxide in exhaled air has a strong correlation to eosinophilic airway inflammation, it is also a compound that cannot be accurately measured by the PTR-ToF-MS [29]. The main reason being that it can be generated within the instrument by ionizing the surrounding air as a primary ion and therefore adding to the general background.  It has long been known that nitrogen oxide plays a role in the anti-viral response of the immune system by creation of some free radicals, but it is unknown exactly how this influences the response to COVID-19 [30]. Since in this case the amount varies systematically between positive and negative patients, the presence and absence can be seen as indicative of a metabolic response to a viral infection. However, the quantitative amount needs to be carefully evaluated on a case-by-case basis and shall not be used as indication on the severity of infection.

The MH study was conducted during week 36 of the influenza season and HF study was conducted during week 46. In both facilities the impact of social distancing and masking measures to the abundance of flu cases has been seen. However, week 36 is for the area of the MH study traditionally an area of low flu incidence and has been equivalent in 2020 to the years before. Week 46 in the area of the HF study has seen a significant decrease in the number of influenza cases. One aspect that needs to be considered is that the metabolic response provides an overlapping response in biomarkers. For example, acetaldehyde is an easily identifiable compound with PTR-MS, but also with GC-IMS, and has been shown to be elevated in exhaled breath for either infection, Influenza A and COVID-19^11,16,31^. This can cause false identifications independent of the incidence rate for Influenza A. This study focused on the development of an algorithm that takes the whole spectral information into account and not only specific compounds of interest. There is not clear evidence in the time being whether the algorithm can distinguish between COVID-19 and Influenza A infected patients.

Variants of COVID-19 were not dominant at the time of the study. In addition, since this study is aiming at metabolic responses and not at viral parts, as a first order assumption the breath pattern change can be assumed to be equivalent for the different variants. External validation and identification of the impact of heightened cases of other diseases in fall-winter 2021, such as RSV or COVID-variants are the next steps to further develop this method.

The main goal of the prediction model was to identify a multiplet of patterns that validate the PCR-based positivity of a patient. To achieve this, the complete mass spectrum was analyzed, and not only the compounds that had obvious changes in their concentration between the average positive and average negative subjects. Apart from nitrogen oxide, compounds identified as important by the model predictions consisted of aldehydes, carboxylic acids, alkenes and alcohols, all of which are common compounds that can be found in human breath. Discrepancies were noticed in the concentration of these compounds in the breath of a healthy and a COVID-19 sick donor. Aldehydes are derived, along with hydrocarbons, from lipid peroxidation and inflammatory processes and have been reported widely in a range of respiratory conditions. Ruszkiewicz and colleagues [16] have also found aldehydes (ethanal, octanal, propanal, heptanal etc.) signals to be elevated in patients with COVID-19 infection. Grassin-Delyle and colleagues [11] identified aldehydes (methylpent-2enal, nonanal), 2-4 octadiene and 1-chloropentane as tracers for COVID-19 infection. While in our case the tested subjects included a variety of hospitalized and non-hospitalized COVID-19 patients, in the Grassin-Delyle and colleagues [13] study all the tested subjects were on mechanical ventilation in the ICU. The differences in the identified compounds (from a direct comparison) can be explained assuming that the progression of physiologic response of ambulatory patients is different compared to that of severely ill and ventilated patients.

In a recently published study, Berna and colleagues [31] reported on the identification of breath biomarkers for children with COVID-19. In their comparative analysis of the compounds with elevated concentrations for infected patients, six compounds were identified as being characteristic: three are aldehydes (octanal, nonanal, heptanal), two are alkenes (dodecane and tridecane) and one is a ring-ether (2-Pentyl-Furan). Isoprene was actively excluded, since it is omnipresent in people's breath at elevated concentrations compared to ambient air, regardless of infection status; our dataset shows the same results, with no indication of the level of isoprene in regard to infection status independent of age.

For nonanal, our study confirms the increase for positive patients as shown in children [31], but we also found a gradual increase in the level of elevation based on age – the older the patients the higher the average elevation. Nonanal was also a key biomarker reported by Grassin-Delyle and colleagues [13] in their adult hospitalized PTR-MS study as is in the study by Ruszkiewcz and colleagues [12]. For octanal, a similar trend was observed as with nonanal.

Acetaldehyde which has been identified in adults [16] to be increased is not discussed in the children's study [31]. We see it as a primary discriminator in the older population with average double increases for people of over age 55, while the younger population showed less of an increase. In addition, acetaldehyde, has been shown to be a biomarker for Influenza A infections [11]. Ketones, such as 2-butanone, have been shown to be elevated in adults [16], but not in children [30] which is also shown in our dataset.

Propionic acid, acetone and hexanal were identified as tracers for Influenza A and Influenza H1N1^5^. Traxler and colleagues [11] have also found acetaldehyde, acetone, propanal and n-propyle acetate to be elevated in patients infected by Influenza A. In our analysis we excluded acetone, since it is a common compound in human breath and is also linked to Influenza A. Even though some of the identified compounds from our study are the same with the corresponding ones for Influenza A infection, we are confident that our approach of using a combination of multiple compounds instead of a few tracers for the determination of COVID-19 infection has the capability of distinguishing the COVID-19 patients from the Influenza A patients.

From the onset of this study, the authors understand that there are several implications that need to be studied further. Such implications include the effect of chronic medical problems in a patient's breath. Moreover, the various medications that patients routinely use can affect their breath pattern[2]. Finally, it is not clear yet if compounds with nitrogen containing elements or those that affect the nitrogen oxide pathway cause either an improvement or detriment to the ability to fight viruses via this mechanism. One additional implication concerns the current antigen-based testing and the unreliability of how long the infected person will shed identifiable antigen [32]. In at least one study [33], 99 of 851 patients continued to test positive by traditional testing greater than four weeks after their first positive. In a meta-review, Henderson and colleagues [32] have well outlined the issue of viral RNA shedding and continued positive antigen testing in conjunction with viral culturing. Their conclusion, while offering sound advice, offers no definitive solution to this issue. We believe that as our method of testing relies upon physiologic change as opposed to direct antigen detection that breath analysis has the potential to determine when the person is no longer at threat of infecting others. This will require further studies including viral cultures to prove definitively.

The presented study's first and foremost goal was to verify whether an algorithm can be found to interpret the mass spectra of breath samples from any random individual, without further knowledge on the circumstances of sampling. All other studies at the point in time of initiation were aims at hospitalized settings or equivalent, therefore requiring a careful evaluation if the breath sample is contaminated by exterior air or not. The goal of finding a pattern of multiple different AI-based algorithms and combination therefore led to the final algorithm that now allows for the identification of COVID-19 in patients without further knowledge on the specific conditions of the sample.

The algorithm in its current form cannot distinguish between symptomatic and asymptomatic positive samples. The authors are planning on expanding the capabilities of the algorithm in order to achieve such separation in future studies by testing more asymptomatic positive patients and study the behavior of the model. Such tests coupled with the corresponding model adjustments will allow a better characterization of the asymptomatic positive samples which could make possible the separation between those two categories.

While this method does not measure the presence or absence of the virus and is therefore not meaningful to determine whether or not a person is infectious through their viral load, it offers the ability to identify whether or not a person is infected and actively fighting off said infection.

The accuracy and speed of such analysis makes it a perfect tool for public health measures in areas where large crowds are anticipated, events of all kinds, or in areas of concentrated continuous presence, such as airplanes.

## Contributors

A.L., H.S., J.W., S.W., performed the measurements. A.L. and A.T. performed the data analysis and drafted the manuscript. P.G.M. developed the model and contributed to the manuscript writing. A.L., A.T., H.H., C.W., R.L. organized both studies and helped with the data interpretation and the manuscript writing. A.L., T.B., K.M., K.B., M.D.F., K.D. collected the samples and organized the study at Henry Ford Hospital. All authors read and approved the final version of the manuscript.

## Data sharing statement

The study protocol and the datasets generated during and analyzed during the current study will be available with publication from the corresponding author on reasonable request.

## Funding

This study was funded by RJ Lee Group Inc. in Monroeville, PA.

## Declaration of Competing Interest

A.L. worked as a consultant for RJ Lee Group. A.L. is employed by RJ Lee Group, who funded all this research. H.H. reported that the study was performed under Personal Service Agreement with RJ Lee Group and received consulting fees from RJ Lee Group. C.W. reports possible profit sharing from RJ Lee Group if commercialised. CW is affiliated with Mercyhealth but no benefit was received. P.M. received support for the current manuscript from RJ Lee Group. S.W. reports that she received 5 Tedlar bags from Restek when the type of bags that would be used in the study was being determined. H.H., R.L., A.T., A.L., J.W., S.W., and C.W. are named as inventors on a patent application covering detection of COVID-19 biomarkers in human breath using mass spectrometry. All other authors have nothing to declare.

## Appendix Supplementary materials

Image, application 1
