# Supplementary material for: A method for the identification of COVID-19 biomarkers in human breath using Proton Transfer Reaction Time-of-Flight Mass Spectrometry
Source: eClinicalMedicine. 2021 Nov 20;42:101207. doi: 10.1016/j.eclinm.2021.101207 (PMC8604657; doi:10.1016/j.eclinm.2021.101207)
Supplement: Supplementary file 1 [file mmc1.docx]

Appendix. Supplementary Material

**A method for the identification of COVID-19 biomarkers in human breath using PTR-ToF-MS**

Aikaterini Liangou MSc, Antonios Tasoglou PhD, Heinz J. Huber PhD, Christopher Wistrom DO, Kevin Brody MD, Prahlad G Menon PhD, Thomas Bebekoski MD, Kevin Menschel DO, Marlise Davidson-Fiedler MD, Karl DeMarco MD, Harshad Salphale, Jonathan Wistrom^7^, Skyler Wistrom and Richard J. Lee PhD

Blank experiments

Blank tests were performed before both studies in order to determine the bag type with the less impurities for our method. Three different bag categories were tested; tedlar, alter and multi-layer bags. Two bags from each category were used for these blank tests. Ultra-pure air (from the same cylinder and at the same time) was injected in all 6 bags and the total VOCs were therebefore measured using the PTR-ToF-MS. The Figure below shows the total VOCs corrected count per second (cps) in each one of these bags. The TEDLAR bags showed to have the least impurities.

**Fig. S1.** Corrected counts per second of the total VOC concentration for three different bag categories. Two bags from each category were tested. Blue bars show the first bag from each category and red bars show the second bag from each category.

Also, the degradation of the total VOCs was monitored in each one of these bags for a total of 2 hours. The Figure below shows the total VOC cps at the time of the injection, 1 hour later and 2 hours later. The results showed that the vapor wall losses were lower in the case of the Tedlar bags.

**Fig. S2.** Total VOC corrected count per second (cps) for each of the three different bag categories. The green bars represent the VOC cps when the bags were filled with ultra-pure air. The red bars show the cps 1 hour after injection and the blue bars the concentration 2 hours after injection.

**Fig. S3**. Schematic of sample pre-processing.

**Fig. S4.** Model development life-cycle overview.

**Fig. S5.** Heatmap of compounds for the total samples collected. The y axis represents the total samples while the x axis represents the m/z 30 to 100. The z axis shows the normalized to the primary ion concentration at m/z 21 (referred as normalized mass). The purple line represents the separation between positive and negative samples. The samples above the purple line are the positives and the samples below the purple line are the negatives.

**Fig. S6.** Abundance of important m/z’s for all the samples collected. The abundance reflects the fraction of the concentration of each compound over the total concentration of the 5 most important compounds The green line represents the separation between positive and negative samples. The samples above the green line are the COVID19 positive and the samples below the green line are the negative.

**Fig. S7.** Abundance of important m/z’s for all the samples over 55 years old collected. The abundance reflects the fraction of the concentration of each compound over the total concentration of the 5 most important compounds. The green line represents the separation between positive and negative samples. The samples above the green line are the COVID19 positive and the samples below the green line are the negative.

**
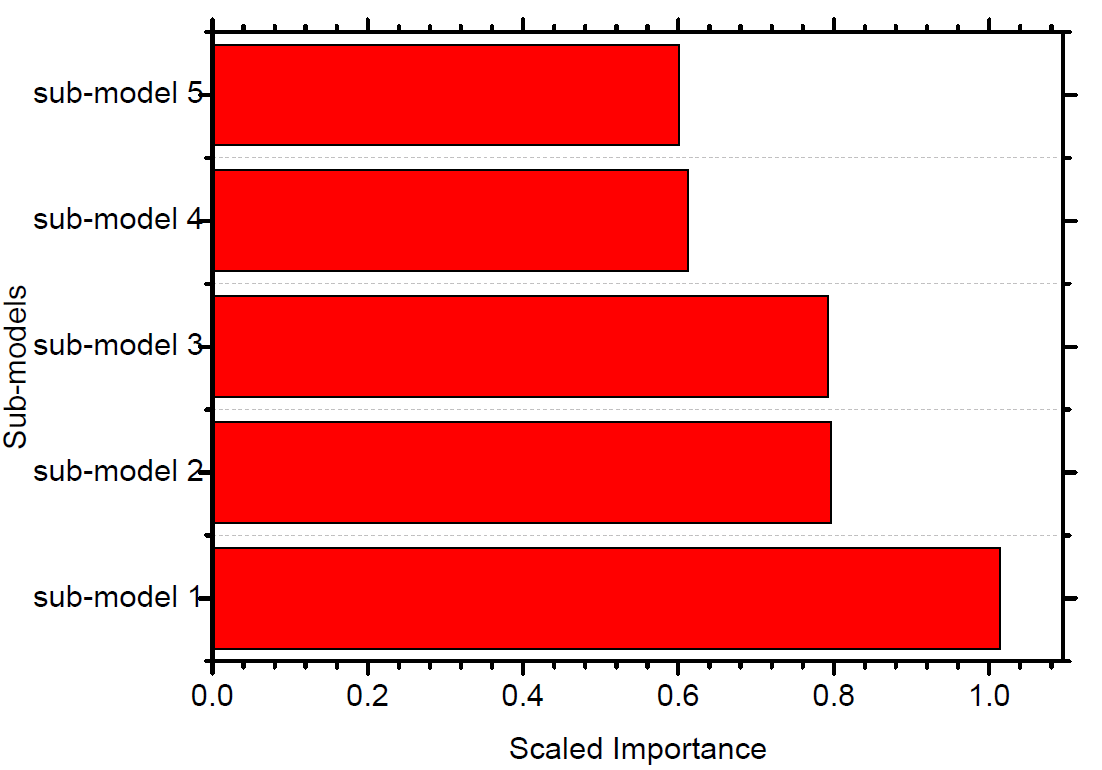
**

**Fig. S8**. Scaled relative importance of each of five sub-models utilized in the determination of a sample being positive or negative to COVID-19. The sub-models adopted from top to bottom were Deep Learning using a fully connected neural network design (submodel 5), Distributed Random Forest (DRF) (submodel 4), XGBoost (submodel 3), Extreme Randomized Trees (XRT) (submodel 2), and Gradient boosting (submodel 1).

**Table S1**. Calculated sensitivity, specificity, accuracy, negative predictive value and precision for model predictions.

|  | **All Age Groups** | **Over 55 years old** |
| --- | --- | --- |
| **Sensitivity** | 81% | 97% |
| **Specificity** | 81.20% | 97.60% |
| **Accuracy** | 81.20% | 97.30% |
| **Negative Predictive Value** | 91.70% | 98.20% |
| **Precision** | 62.60% | 97.60% |

**
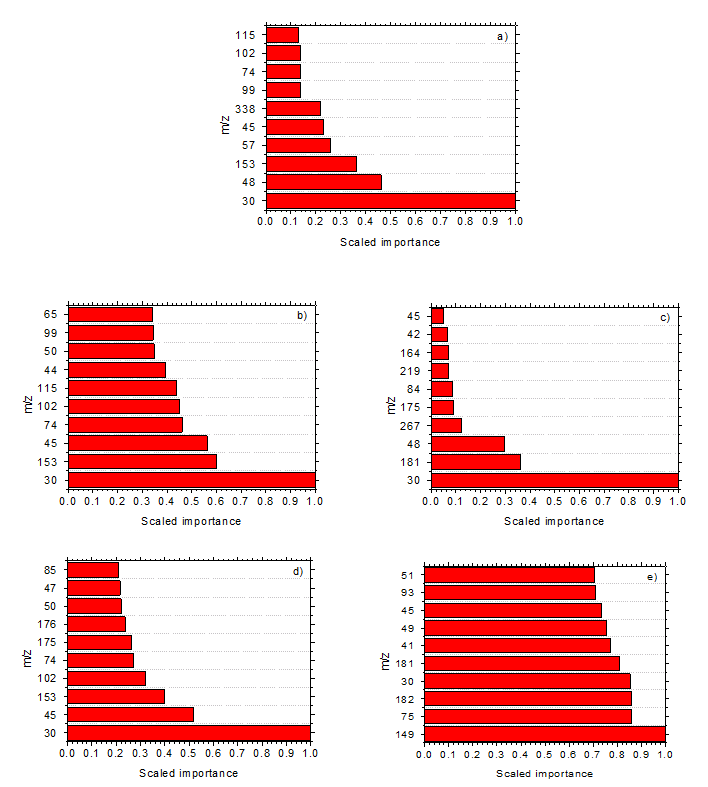
**

**Fig. S9.** The ten most important compounds for the prediction of COVID-19 for each submodel, identified by a) Gradient boosting, b) Extreme Randomized Trees (XRT), c) XGBoost, d) Distributed Random Forest (DRF) and e) Deep Learning using a fully connected neural network design. The y-axes show the m/z ratios while the x-axes show the scaled importance of the compounds within each submodel.

**Table S2**. Results from model using normalized mass spectra.

| **Measured** | **Predicted**  **Negative** | **Predicted positive** | **Grand Total** |
| --- | --- | --- | --- |
| Asymptomatic COVID19 | 27 | 0 | 27 |
| Symptomatic COVID19 | 43 | 29 | 72 |
| Negative | 247 | 0 | 247 |
| **Grand Total** | **317** | **29** | **346** |

**Table S3.** Selected biomarkers comparison. Reported increase is average amount for PCR-positive over average amount within PCR-negative patients for the three age groups of 55+, less than 55 and less than 40 years. Compounds were chosen on the basis of having been reported by either Ruskiewicz and colleagues (Rus), Berna and colleagues (Ber) or Grassin-Delyle and colleagues (Gra). Asterisk reminds that Berna and colleagues did not see a significant increase in ketone concentrations within pediatric patients with SARS-CoV-2 infection.

| **Compound** | **Formula** | **Mass** | **Reference** | **Increase in [%] for PCR-positive over negative** | | |
| --- | --- | --- | --- | --- | --- | --- |
|  |  |  |  | **age >55** | **age <55** | **age <40** |
| Acetaldehyde | C_2_H_4_O | 44.05 | Rus | 75 | 19 | 5 |
| Acetone | C_3_H_6_O | 58.08 | Rus/Ber* | 31 | 46 | 47 |
| 2-Butanone | C_4_H_8_O | 72.11 | Rus/Ber* | 53 | 9 | 6 |
| Methylpent-2-enal | C_6_H_10_O | 98.14 | Gra | 69 | 12 | -2 |
| 2,4-octadiene | C_8_H_14_ | 110.20 | Gra | 9 | -3 | -9 |
| Heptanal | C_7_H_14_O | 114.19 | Rus/Ber | 7 | 28 | 39 |
| Octanal | C_8_H_16_O | 128.21 | Rus/Ber | 44 | 9 | -1 |
| 1-chloroheptane | C_7_H_15_Cl | 134.65 | Gra | 18 | 10 | 0 |
| 2-pentyl furan | C_9_H_14_O | 138.21 | Ber | 10 | -10 | -10 |
| Nonanal | C_9_H_18_O | 142.24 | Gra/Ber | 44 | 23 | 10 |
